# Endoscopically assisted removal of ectopic teeth in the floor of the orbit. Case report and latest literature review

**DOI:** 10.4317/jced.61371

**Published:** 2024-05-01

**Authors:** Imanol Zubiate-Illarramendi, Fernando Monsalve, Paolo Cariati, Carmen Camacho-Sanchez-Mora, Blas García-Medina

**Affiliations:** 1 MD. Resident. Hospital Universitario Virgen de las Nieves, Granada. Spain. Oral and Maxillofacial Surgery Department; 2 MD., Professor. Hospital Universitario Virgen de las Nieves, Granada. Spain. Oral and Maxillofacial Surgery Department

## Abstract

Ectopic sinonasal third molar is a rare condition characterized by the aberrant position of the third molar in the maxillar sinus. The etiology of the teeth in the maxillary sinus is commonly associated to trauma and iatrogenic dental procedures. We present the clinical case of a 33-year-old man who presents an ectopic tooth in the right maxillary sinus, located in the orbital floor, who requires endoscopic control through a maxillary approach when performing the extraction. The endoscopic technique is being increasingly used in the maxillofacial field. Thanks to the endoscopic control for complex extractions like the descibed case, complications such as injuries of the infraorbital nerve or fractures of the different walls of the sinus could be avoided. In conclusion, endoscopic control to perform tooth extractions in the maxillary sinus is a safe option that helps to avoid complications such as fracture of the maxillary sinus walls or fracture of the orbital floor.

** Key words:**Endoscopic, ectopic tooth, caldwell luc, maxillary sinus.

## Introduction

Ectopic sinonasal third molar is a rare condition characterized by the aberrant position of the third molar in the maxillar sinus. The etiology of the teeth in the maxillary sinus is commonly associated to trauma and iatrogenic dental procedures. Nevertheless, supernumerary teeth can also erupt idiopathically into the sinus or nasal cavity (1).

Caldwell-Luc approach is the most commonly used maxillar approach to remove foreign bodies and teeth from the maxillary sinus. However, different sinus and nasal foreign bodies can be removed by an approach through the nasal antrum. The extraction of teeth from the sinus is a high level surgery because it requires osteotomy of the anterior wall of the sinus and the subsequent extraction of the tooth, with the risk of fracturing the medial, lateral or posterior wall of the maxillary sinus (2).

The clinical presentations of sinonasal teeth are nasal osbtruction, facial pain, rhinorrhea, epistaxis, foul odor sensation, sinusitis, orosinus fistula and hyposmia (3).

The most used imaging tests for the diagnosis of this pathology are orthopantomography and CT SCAN. Sometimes a simple orthopantomography is enough to locate and extract the tooth, but a 3D image using CT SCAN could be essential to avoid the injury of the infraorbital nerve and the different walls of the maxillary sinus (4).

We present the clinical case of a patient who presented the upper right third molar (18) in an atypical location such as the floor of the orbit, requiring endoscopic control when performing the extraction.

## Case Report

We present the case of a 33-year-old man who attended maxillofacial consultations due to an orosinus fistula in the right maxilla with episodes of suppuration and facial pain during 3 weeks.

In the intraoral exploration, a fistula was observed in the right maxillary vestibular mucosa, which was painful on palpation.

**Figure 1:**
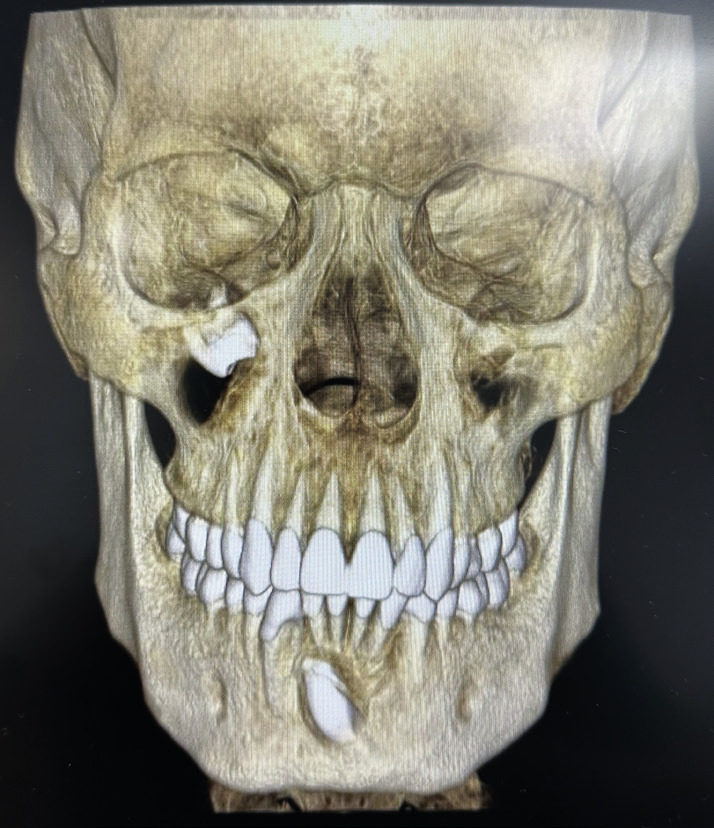
3D image of the CT SCAN showing the ectopic wisdom tooth located in the right orbital floor, within the maxillary sinus.

Orthopantomography and CT SCAN were performed for the diagnosis. In both images, ectopic tooth (18) could be seen located in the right maxillary sinus. After viewing the images on the CT SCAN in 3D, the existence of a right intrasinus cyst with tooth 18 in the right orbital floor was confirmed (Fig. 1). As the patient presented symptoms of infection during several weeks, the extraction and cystectomy was chosen with endoscopic visualization.

**Figure 2:**
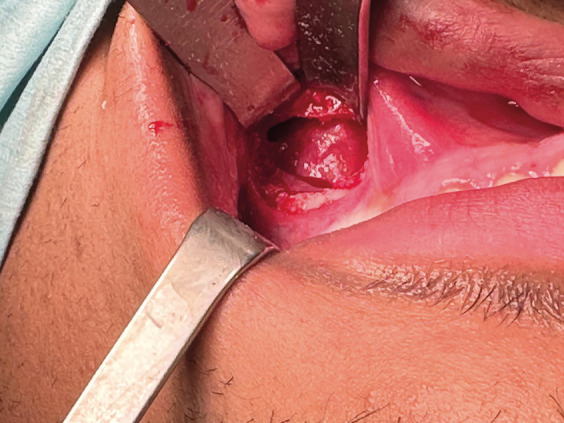
Caldwell-Luc approach in the right maxilla, showing the cyst that retains the ectopic tooth.

**Figure 3:**
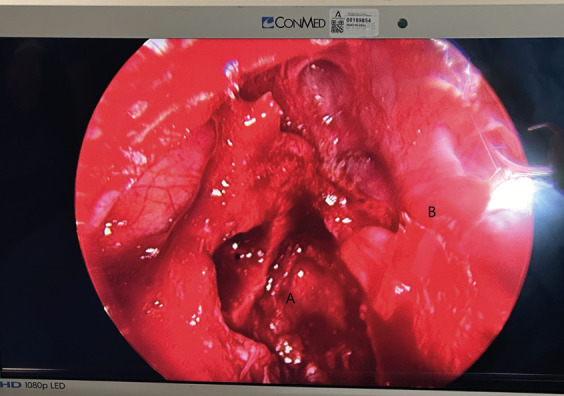
Image observed after introducing the 30º endoscope through the maxillary approach. A (orbital floor, observing the place where the ectopic tooth was located), B (medial wall of the maxillary sinus).

Under general anesthesia with left nasotracheal intubation. An incision was made in the right vestibular mucosa from teeth 12 to 16. Using a Caldwell-Luc approach in the right vestibular mucosa, the cystic cavity within the maxillary sinus was visualized with the presence of the included ectopic molar (Fig. 2). Due to the risk of fracturing the orbital floor when performing the extraction, it was decided to perform it under endoscopic control through the maxilla using the Caldwell-Luc approach. A 30º endoscope was used and the integrity of the orbital floor and the rest of the walls of the maxillary sinus were checked after removing the tooth and cystectomy was done (Fig. 3).

**Figure 4:**
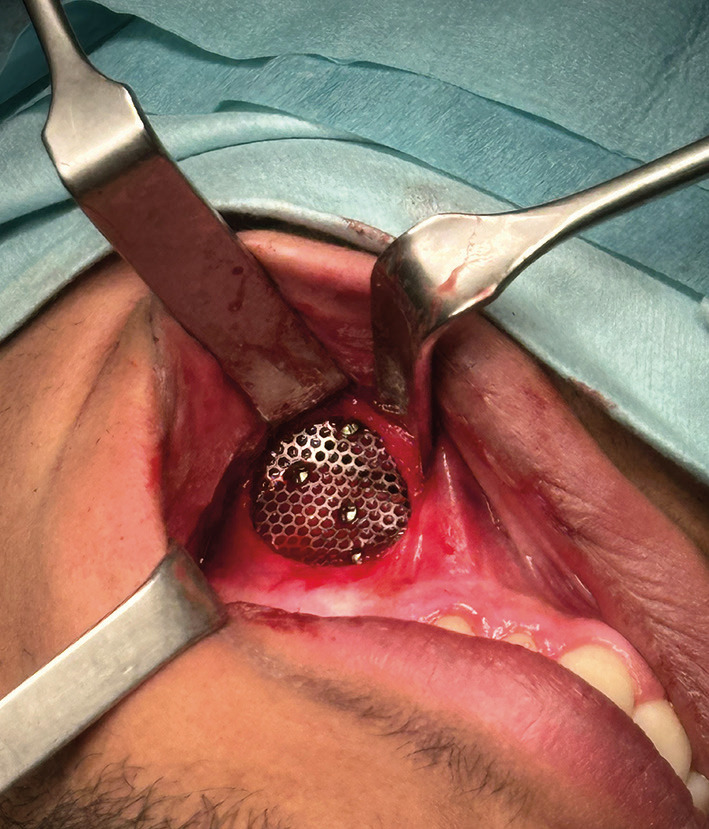
Closure of the surgical approach with the placement of the titanium mesh fixed with 4 screws.

Titanium mesh fixed with four 4mm screws was placed on the anterior wall of the maxillary sinus to cover the defect left by the maxillary approach (Fig. 4). Direct closure with 4/0 vycril of the nasal mucosa was performed. The patient went home 12 hours after the surgical intervention and had no postoperative complications.

The patient was reviewed 3 and 12 months after surgery and had no local complications. There was not exposure of the mesh placed on the anterior wall of the maxillary sinus and there was not anesthesia of the infraorbital nerve. The pathological anatomy result of the cyst study was a follicular cyst.

## Discussion

The ectopic teeth in the maxillary sinus are not common are there are very few cases reported in the Oral and Maxillofacial literature. The most common reason for a maxillary sinus cavity foreign body or teeth is a dental procedure. Furthermore, supernumerary teeth in the maxillary sinus only occur in 0.1-1% of the general population. The etiology of a ectopic teeth may be attibuted to the primary dislocation caused by deviant position of teeth germs, aberrant eruption pattern and lack of space for third molars, or the secondary ectopic dislocation such as trauma, infection, cyst and tumors (5).

Among these patients, most frequent symptoms are facial pain, orosinusal fistula, suppuration, sinusitis, skin erythema, orosinusal communication and infraorbital nerve anesthesia (3).

Due to the complexity of this type of extraction, the radiographic study is essential to be able to carry out a correct planning for dental extraction. The first test to be performed should be an orthopantomography. Subsequently, performing a CBCT or a CT SCAN would be the gold standard for a more precise diagnosis (6). Thanks to 3D reconstruction, the ectopic tooth could be exactly located in the maxillary sinus and this would help to plan the surgical approach for extraction more precisely, in addition to avoid possible risks or complications like infraorbital nerve injury. In the described case, 3D reconstruction and CT SCAN study were essential to locate the ectopic tooth on the orbital floor and assess the risks of surgery (6,7).

There are different types of approaches for the extraction of ectopic teeth. If they occur in the mandibular ramus or near the condyle, the approach could be extraoral or intraoral. The extraoral approach produces a greater vision of the surgical field but presents risks of facial nerve injury, salivary fistula and skin scar. The intraoral approach is safer, with fewer complications, but with a smaller field of visión (8). In the case of ectopic teeth in the maxillary sinus, the most used approach is the Caldwell-Luc because an adequate field of vision is achieved through an osteotomy of the anterior wall of the maxillary sinus and the risk of complications is minimal (9).

Endoscopy is a growing technique that is being increasingly used by otorhinolaryngology, since it provides a minimally invasive approach to the maxillary sinus to treat different pathologies such as sinusitis, foreign body extraction or excision of lesions. In cases of ectopic teeth, endoscopic control could be essential to achieve a minimally invasive approach and control at all times that no injuries to the walls of the maxillary sinus or fracture of the orbital floor occur (10). Liau *et al* (11) reported the use o fan endoscopically assised intraoral approach for extraction of ectopic mandibular third molar with no obvious nerve injury.

Dental extractions through endoscopic control are performed very occasionally but it is a way to perform them safely, since with the advances in technology, minimally invasive techniques are being increasingly used with the aim of reducing complications and hospital stay.

## Conclusions

Endoscopic control to perform tooth extractions in the maxillary sinus is a safe option that helps to avoid complications such as fracture of the maxillary sinus walls or fracture of the orbital floor. Nevertheless, more cases should be published to know the real advantages of endoscopically controlled extraction.

## Data Availability

The datasets used and/or analyzed during the current study are available from the corresponding author.
